# Isolation, Characterization, and Genome Engineering of a Lytic *Pseudomonas aeruginosa* Phage

**DOI:** 10.3390/microorganisms12112346

**Published:** 2024-11-16

**Authors:** Xiaomei Cong, Shuang Zhao, Qing Zhang, Shuo Liu, Youming Zhang, Fu Yan

**Affiliations:** 1 State Key Laboratory of Microbial Technology, Shandong University, Qingdao 266237, China; 13371370369@163.com (X.C.); zhaoshuang122011@163.com (S.Z.); 202212520@mail.sdu.edu.cn (S.L.); 2 Institute of Animal Science and Veterinary Medicine, Shandong Academy of Agricultural Sciences, Jinan 250100, China; zhangqingchina@163.com

**Keywords:** bacteriophage, *Pseudomonas aeruginosa*, *Phikmvvirus*, genome reduction, genome engineering

## Abstract

Antibiotic-resistant bacterial infections have become one of the leading causes of human mortality. Bacteriophages presented great potential for combating antibiotic-resistant infections in the post-antibiotic era due to their high host specificity and safety profile. *Pseudomonas aeruginosa*, an opportunistic pathogenic bacterium, has shown a surge in multidrug-resistant strains, severely impacting both human health and livestock. In this study, we successfully isolated and purified a *P. aeruginosa*-specific phage, PpY1, from feces collected from a breeding farm. This phage harbors a short tail and a 43,787 bp linear genome, and exhibited potent lytic activity against several pathogenic *P*. *aeruginosa* strains. Leveraging Transformation-associated recombination (TAR) cloning and phage assembly techniques in a *P. aeruginosa* host lacking a restriction–modification system, we developed a genome engineering platform for PpY1. Through a systematic gene knockout approach, we identified and eliminated 21 nonessential genes from the PpY1 genome, resulting in a series of phages with reduced genomes. This research not only enhances our understanding of the phage genome but also paves the way for the functional optimization of phages, e.g., broadening the host spectrum and elevating the lytic capacity, dedicated towards the treatment of bacterial infections.

## 1. Introduction

Infectious diseases have historically exacted a significant toll on human society. The advent and subsequent development of antibiotics marked a pivotal milestone in the treatment of bacterial infections, saving countless lives. However, the extensive use and misuse of these drugs have contributed to the alarming rise in antibiotic-resistant bacteria. Antibiotic resistance is now recognized as a grave threat to global health, posing a formidable challenge to the efficacy of medical treatments [1]. The spread of multidrug-resistant bacteria, especially the ‘ESCAPE’ pathogens (*Enterococcus faecium*, *Staphylococcus aureus*, *Klebsiella pneumoniae*, *Acinetobacter baumannii*, *Pseudomonas aeruginosa*, and *Enterobacter* spp.), has become a critical public health concern [2,3]. Without sufficient action, antimicrobial resistance is estimated to cause approximately 10 million deaths each year by 2050 [4]. Bacteriophages are viruses that specifically infect bacteria, which exhibit great diversity and are ubiquitous on earth [5,6]. They play crucial roles in ecosystems and can be isolated from a variety of environments wherever their host bacteria reside [7]. Phages have garnered significant attention for their potential applications, including advancing our understanding of molecular biology and genetics [8,9,10], serving as vectors for DNA transfer [11,12], providing diagnostic tools [13], and emerging as innovative therapeutic agents [14]. While bacteriophages are known to infect bacteria, they could be classified as temperate and lytic phages. Temperate phages could integrate their genomes into host genomes and carry the risk of transferring antibiotic resistance or pathogenicity islands to bacteria. On the other hand, lytic phages induce host cell lysis upon infection through the lytic replication cycle and are preferred in phage therapy [15].

*Pseudomonas aeruginosa* is a notorious opportunistic pathogen that triggers a spectrum of acute and chronic infections in humans and animals. It has become a major cause of nosocomial infections and a prime exemplar of antibiotic resistance [16]. Multidrug-resistant *P. aeruginosa* strains are highly concerning, particularly for the immunocompromised patients and those with cystic fibrosis (CF) [17,18]. The ability of *P. aeruginosa* to form biofilms and its fast-developing resistance to antibiotics complicate treatment, necessitating the search for alternative therapeutic strategies. Phage therapy has emerged as a promising strategy for combating *P. aeruginosa* infections [19]. Although phage therapy has shown promise in treating *P. aeruginosa* infections [20,21], its effectiveness can be constrained by a narrow host range and bacterial defense mechanisms, such as CRISPR-Cas and restriction–modification (RM) systems. However, there is a possibility of solving these challenges through synthetic biology approaches.

By manipulating receptor-binding proteins and their relevant structural domains, it is possible to broaden the host range and bypass resistance [22,23]. Cutting-edge technologies, e.g., the CRISPR-Cas system, have accelerated the genetic modification of phage genomes [24,25]. However, resulting from restrictions to genetic tractability and phage-defensive systems in host cells, the toxicity of phage proteins, and our limited understanding of phage genomes, etc., the genetic manipulation of complicated phage genomes and rebooting of phages remain quite challenging.

Yeast has a significant advantage in phage genome assembly due to its significant genetic differences from bacteria and a powerful DNA recombination system. In a yeast like *Saccharomyces cerevisiae*, the toxicity caused by phage proteins could be avoided [26], and transformation-associated recombination (TAR) cloning allows for the assembly of DNA fragments with homologous arms less as 60 bp with high efficiency [27,28]. By using this method, several *P. aeruginosa* phages were rebooted from the assembled genome [29], and genome scaffolds of *Escherichia coli* and *Klebsiella* phages were engineered, leading to phage tail components being swapped with target hosts [30]. In addition, phages can be reprogramed as vectors to deliver antimicrobial proteins to bacteria [10,31]. However, due to the limitation of viral particle’s capability to encapsulate DNA [32,33], it is necessary to minimize the size of phage genomes to accommodate all of the antibacterial genetic elements. Despite great advances in bioinformatics technology, the functions of most phage genes remain unknown, with about 70% of their products being annotated as “hypothetical protein” [34]. These genes may enhance phages’ ability to adapt to diverse ecological niches, but they might not be essential for phage development under certain conditions [35].

In this research, we successfully isolated a lytic bacteriophage, designated as PpY1, from fecal samples, targeting *P. aeruginosa*. The robust lytic activity of PpY1 against several *P. aeruginosa* strains from both human and animal origin provided an ideal model for phage engineering. Using next-generation sequencing technology, we determined the genome sequence of PpY1. Following the assembly of the PpY1 genome in yeast and subsequent reactivation within *P. aeruginosa*, we conducted a comprehensive gene knockout study. This systematic approach led to the identification of 21 dispensable genes and the development of a suite of phage variants with reduced genomes. The findings of this study are pivotal for uncovering the functions of previously uncharacterized phage genes and for the strategic enhancement of phage therapeutics.

## 2. Materials and Methods

### 2.1. Nucleotides and Bacterial Strains

All oligonucleotides used in this study were synthesized by Sangon Biotech (Table S1). All plasmids constructed and used in this study are listed in Table S2. *P. aeruginosa* was cultivated in LB liquid medium at 37 °C. *S. cerevisiae* VL6-48 was propagated in YPDA (YPD medium supplemented with adenine) at 30 °C. The plasmid pBBR1-Rha-redγ-BAS-kan [36] was transformed into *P. aeruginosa* PAO1 by electroporation, yielding the strain PAO1-γBAS. The gentamicin resistance gene *gentaR* with 120 bp homology arms of RM system [37] genes (locus-tag: PA2732-PA2735; genes *PaePAIP*-*M. PaePAIP*) was amplified by PCR using the primers genta-KORM-F1/genta-KORM-R1 and genta-KORM-4-F2/genta-KORM-4-R2 (Table S1), followed by transferring it into recombinase-proficient PAO1-γBAS. The RM system was then substituted with *gentR* through homologous recombination [36,38]. The RM system-deficient strain was designated as PAO1-KORM.

### 2.2. Phage Screening and Purification

Phages specific to *P. aeruginosa* PAO1 were isolated from fecal samples collected from Jimo Jiarun Farm (a pig farm) in Qingdao, China. The fecal samples were centrifuged at 1000× *g* for 10 min. The supernatants were filtered through a 0.22 µm syringe-driven filter, mixed with 50 mL of PAO1 cells at log-phase, and incubated at 37 °C overnight. The cultures were centrifuged and filtered and the phages were then cultured using the double-layer agar plate method. Single plaques were picked up using a sterile toothpick and saved in SM buffer (100 mM NaCl, 8 mM MgSO_4_·7H_2_O, 50 mM Tris-HCl, pH 7.5). Multiple rounds of purification were performed until the morphology and size of the plaques were stable. Phages were serially diluted in 100 μL LB, and then mixed with 100 μL of overnight bacteria (2.5 × 10^9^ cells per mL) and 7 mL of LB soft agar, and coated on LB agar plates. After incubating at 37 °C overnight, the morphology and size of plaques were analyzed.

### 2.3. Transmission Electron Microscopy

The phage was spotted on a carbon-coated copper grid and negatively stained with 1% (*w*/*v*) phosphotungstic acid. Electron micrographs were captured using a TECNAI G^2^ F20 transmission electron microscope operated at an acceleration voltage of 80 kV.

### 2.4. One-Step Growth Experiment

During the exponential phase, 10 μL of host cells (~2.5 × 10^9^ cells per mL) were infected with 10 μL phages at a multiplicity of infection (MOI) of 0.01 in 1.8 mL of LB medium at 37 °C. The supernatants of 10 µL were withdrawn from the culture mixture at 5 min intervals, extending up to 70 min. The supernatants were then serially diluted and plated to determine the phage titer.

### 2.5. Characterization of Temperature and pH Tolerance

To evaluate the impact of temperature on the stability of phage PpY1, the phage stock solution (~10^12^ PFU/mL) was incubated at a range of temperatures (20, 30, 40, 50, 60, 70 °C) for 1 h. In parallel, to assess the pH stability of the phage, LB medium was adjusted to specific pH levels (3, 5, 7, 9, 11, and 13) using either NaOH or HCl. Subsequently, phage PpY1 (~10^13^ PFU/mL) was added to the medium and incubated at 37 °C for 1 h. The host cells (~2.5 × 10^9^ cells per mL) were cultivated in LB medium, and the phage titer post-incubation was determined using 100 μL of phages and 100 μL of host cells using the double-layer agar method. Independent experiments were repeated thrice.

### 2.6. Host Range Determination

*P. aeruginosa* isolates (Table 1), sourced from the Shandong Academy of Agricultural Sciences, were used as hosts for assessing the antibacterial activity of the phage. For the spot test, 200 μL of freshly cultured bacteria was mixed with 7 mL of 0.7% top agar and evenly spread onto a 1.5% LB agar plate. Once the agar had solidified, 10 μL of the phage suspension (10^13^ PFU/mL) was applied to the surface of the plates and incubated overnight at 37 °C.

### 2.7. Phage Genome Extraction

Lysates were prepared by infecting 50 mL logarithmically growing cells with the corresponding phage until the cultures achieved clearance. DNaseI (Thermo Fisher Scientific, Waltham, MA, USA) and RNaseA (Thermo) were added to the lysates at a final concentration of 1 μg/mL, and incubate at 30 °C for 30 min. Subsequently, 2.92 g of solid NaCl was added to achieve a final concentration of 1 M, and the lysates were stirred until completely dissolved. The mixture was then placed in an ice bath for 1 h. Cell debris were removed by centrifugation at 10,000× *g* for 10 min (4 °C) and filtration through 0.22 μm filters. PEG 8000 was added to the supernatant and the mixture was stored at 4 °C overnight. Samples were centrifuged at 10,000× *g* for 10 min at 4 °C, and the resulting phage pellets were suspended in 800 μL SM buffer. Then, 25 μL proteinase K (20 mg/mL) and 50 μL 10% SDS were added to 400 μL of resuspended phage pellets, and the mixture was inverted gently. The samples were then incubated at 50 °C for 1 h, with inversions until the solution became clear. Next, 650 µL of DNA extraction solution (phenol: chloroform: isoamyl alcohol = 25:24:1) was added, and the mixture was gently inverted until fully emulsified. The emulsion was centrifuged at 8300× *g* for 30 min. The 500 μL upper aqueous phase was carefully transferred to a 2 mL Eppendorf tube, and 35 μL 3 M sodium acetate (pH 7.5) was added, followed by gentle inversion. To precipitate the DNA, 1.2 mL of anhydrous ethanol was added and gently mixed until white flocculent DNA precipitates formed. The DNA was then transferred to 1.5 mL containing 75% ethanol. After centrifugation at 8000× *g* for 3 min, the pellet was air-dried at room temperature. The DNA wad finally dissolved in ddH_2_O.

### 2.8. Bioinformatics Analysis of Phage Genome

The genomic DNA of phage PpY1 was sequenced using the Illumina NovaSeq and Oxford Nanopore ONT techniques (Shanghai Personalbio Technology, Shanghai, China). The gene functions in phage PpY1 genome were annotated using the Rapid Annotation using Subsystem Technology (RAST) server (https://rast.nmpdr.org/ accessed on 12 October 2024) with the RASTtK scheme. The genome similarity and protein functions were analyzed with the BLAST algorithm against the nonredundant nucleotide and protein databases (http://www.ncbi.nlm.nih.gov/BLAST/ accessed on 12 October 2024).

### 2.9. Genome Assembly in Yeast

*S. cerevisiae* VL6-48 was grown in 1.4 mL YPD at 30 °C overnight. Overnight cultures were transferred into 50 mL YPD, and cultivated at 30 °C for 4–6 h. Cells were harvested by centrifugation at 1800× *g* and washed with 50 mL ddH_2_O. Then, cells were washed with 50 mL 1 M sorbitol, and suspended with 20 mL SPEM buffer (1 M sorbitol, 6 mM CaCl_2_, 2.5 g per liter yeast extract and 5 g per liter Bacto peptone). To the mixture, 40 μL zymolyase-20T and 30 μL β-mercaptoethanol were added and mixed well. After incubation at 30 °C for 30 min, 1 M sorbitol was added to the mixture to a total of 50 mL, and centrifuged at 600× *g* for 10 min. The pellet was then resuspended in 50 mL 1 M sorbitol, and was centrifuged at 600× *g* for 10 min. Then, 1.8 mL STC buffer was then used to resuspend cells for transformation. The phage fragments were obtained by PCR using primers with 59 bp homologous arms (Table S1). The PCR products and linear pCAP01 vector DNA were mixed with a 200 μL protoplast of yeast and incubated at room temperature for 10 min. Then, 800 μL 20% PEG 8000 (Sangon Biotech, Shanghai, China) was added and mixed well. After incubation at room temperature for 20 min, the mixture was centrifuged at 700× *g* (4 °C) for 10 min, resuspend in 800 μL SOS buffer, and incubated at 30 °C for 30 min. Transformants were selected on agar plates with complete synthetic defined medium without tryptophan (SD-Trp) at 30 °C for 3 days. Yeast colonies were picked with loops or 200 μL pipette tips and resuspended with 10 mM Tris-HCl (pH = 8.0) containing 0.2 mg/mL zymolyase-20T. After incubation at 30 °C for 2 h followed by boiling for 5 min, the cell debris was removed by centrifugation and the supernatant was used as template for PCR verification.

### 2.10. Plasmid Extraction from Yeast Cells

The transformant yeast colony was inoculated into 15 mL SD-Trp liquid medium and cultivated at 30 °C with shaking for 24–48 h. The cells were harvested and suspended in 1 mL SPEM buffer. Then, 50 μL zymolyase-20T and 1 μL β-mercaptoethanol were added and the mixture was incubated at 30 °C for 1–2 h. P2 solution (TIANGEN, Beijing, China) was then added and the tube was inverted to mix well. Subsequently, P3 solution (TIANGEN) was added to the tube and mixed well. After centrifugation at 8300× *g* for 10 min, the supernatant was transferred to a new tube and 3 mL DNA extraction solution (phenol–chloroform–isoamyl alcohol = 25:24:1) was added. The step was repeated, and then the supernatant (600 μL) was transferred to a new 1.5 mL tube, 400 μL isopropanol was added to the supernatant to precipitate the DNA. The precipitate was washed with 1 mL 75% ethanol, dried at room temperature, and dissolved in 100 μL ddH_2_O. Then, 2 μL of 10 mg/mL RNaseA was added to DNA solution and incubated at 37 °C for 10 min. Subsequently, 4 μL of 5 M NaCl and 70 μL isopropanol were added to the mixture and centrifuged at 13,000× *g* for 20 min. The pellet was washed with 1 mL 75% ethanol, dried, and dissolved in 30 μL of ddH_2_O.

### 2.11. Transferring Phage Genome into P. aeruginosa

*P. aeruginosa* cells grown during the logarithmic growth phase were washed twice using 1 mL of 100 mM sucrose (4 °C) and resuspended in 50 μL of 100 mM sucrose solution. Then, 10 μL of plasmid extracted from yeast was mixed with the bacterial cells and transferred to a 2 mm electroporation cuvette (Bio-Rad, Hercules, CA, USA). Immediately the cells were electroshocked at 2500 V (Eppendorf Eporator, Hamburg, Germany). Cells were recovered in 1 mL of LB broth at 37 °C for 1 h, mixed with 7 mL of LB soft agar (LB with 0.7% agar) and spread on LB agar plates. The plates were incubated at 37 °C overnight.

### 2.12. Antibacterial Assays of Mutant Phages

*P. aeruginosa* cultures in the log-phase were adjusted to an OD_600_ of 0.05–0.1 in LB medium. These cultures were then distributed into clear 100-well plates (Bioscreen II, Shanghai, China) and infected with the phages to achieve a final concentration of 8 × 10^5^ PFU/mL. The plates were subsequently placed into a fully automatic microbial growth curve analyzer (Bioscreen C° Pro, Shanghai, China) and OD_600_ was measured every 5 min at 37 °C. The medium without bacteria served as a negative control to ensure sterility, while uninfected bacteria acted as positive control to monitor normal growth. Each experiment was performed three times.

### 2.13. Statistical Analysis

Statistical analysis was conducted using Microsoft Excel 2019. The results were presented as means ± standard deviation (SD). The significance level was set at *p* < 0.05 (*).

## 3. Results

### 3.1. Characterization of Isolated Phage PpY1

**Figure 1:**
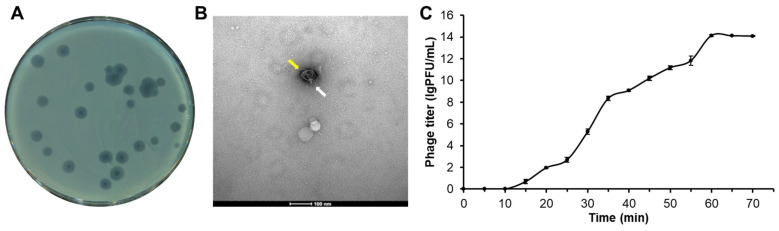
Characterization of phage PpY1. (**A**) Morphology of phage plaques on a double-layer agar plate; (**B**) TEM image of phage PpY1, with a scale bar presenting 100 nm; (**C**) one-step growth curve of phage PpY1, showing average phage titers from three independent cultures. Error bars indicate the standard deviation (SD). Phage head and tail are indicated with yellow and white arrows, respectively.

The bacteriophage PpY1 was isolated from a fecal sample obtained from a breeding farm using the double-layer agar plate technique. The incubation of PpY1 with *P. aeruginosa* PAO1 produced distinct bright plaques surrounded by halos on soft agar plates (Figure 1A). The observations made using with transmission electron microscopy (TEM) revealed that PpY1 possesses a head with a diameter of approximately 60 nm and a tail measuring nearly 20 nm in length (Figure 1B). The one-step growth curve analysis indicated that PpY1 has a latent phase of approximately 15 min, followed by a burst phase lasting 45 min. The phage titer reached a plateau at around 10^13^ plaque-forming units (PFU) per milliliter after 60 min. During the exponential phase, the phage titer increased from 10^2^ to 10^13^ PFU/mL (Figure 1C).

### 3.2. Stability of Phage PpY1 Under Varied pH and Temperature Conditions

**Figure 2:**
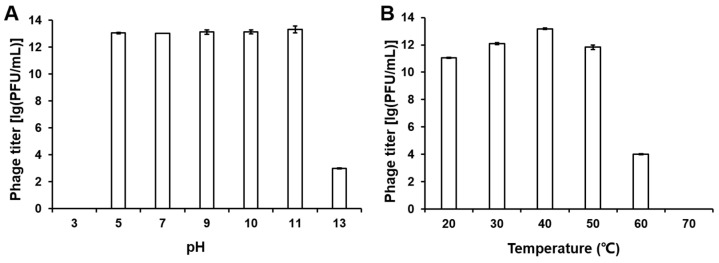
Titer of phage PpY1 under varied pH and temperature. (**A**) Titer of PpY1 under diverse pH; (**B**) Titer of PpY1 at different temperatures. Error bars indicate the standard deviation (SD).

To evaluate the stability of phage PpY1 across a range of environmental conditions, we exposed it to various temperatures and pH levels and monitored the resulting phage titer. Figure 2 illustrates that the phage maintained significant biological activity at pH range between 5 and 7. An apparent decline in phage titer was observed following exposure to pH 13, and no plaques were detected after treatment at pH 3. The phage titer remained relatively stable across temperatures ranging from 20 to 50 °C, with an optimal temperature for phage activity at 40 °C. The titer of the phage experienced a significant reduction at 60 °C, and no plaques were observed at 70 °C.

### 3.3. Host Range of Phage PpY1

The host range of phage PpY1 was determined by exposing it to a panel of 26 different *P. aeruginosa* strains isolated from various environments. Our findings revealed that the PpY1 exhibited potent lytic capabilities against nine of these strains, while it showed a weak lytic effect on one additional strain (Table 1). The susceptible strains, which were derived from a broad spectrum of origins, including animal tissues, feedstuffs, human clinical samples, highlight the versatility of phage PpY1 in combating *P. aeruginosa* infection, including those that are resistant to traditional antimicrobial treatments.

### 3.4. Analysis of the PpY1 Genome

**Figure 3:**
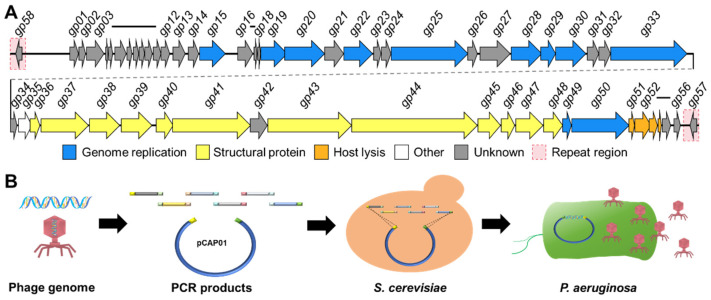
Analysis and assembly of phage PpY1 genome. (**A**) Genome structure of phage PpY1; (**B**) diagram of phage genome assembly and rebooting.

Sequencing of the PpY1 genome resulted in the assembly of a linear genome in a size of 43,787 bp with an average guanine-cytosine (G+C) content of 62.2% (Genbank assession number: PQ463998). This genome is characterized by two direct terminal repeats (DTRs) of 440 bp at both ends. Employing the Rapid Annotation using Subsystem Technology (RAST) and Basic Local Alignment Search Tool (BLAST) analyses, we predicted 58 ORFs encoding proteins with at least 30 amino acids as potential genes (*gp01*-*gp58*, Figure 3). Among these, 27 gene products showed a significant homology to proteins with known functions. The remaining 30 genes, which did not show a significant homology to any known proteins, are hypothesized to encode hypothetical proteins. A thorough examination of the PpY1 genome did not reveal any lysogeny modules or lysogens (Table 2), confirming that phage PpY1 is indeed lytic in nature. BLASTn analysis of the complete genome sequence revealed that the isolated phage PpY1 shared a high similarity with the well-known *P. aeruginosa* phage φKMV with nucleotide sequence identity of 91.6% [39]. The genome arrangement of PpY1 mirrors that of φKMV with the phage RNA polymerase gene situated in the central region of the genome. We also identified several T7-like tail components within the PpY1 genome, including T7-like proteins like head-to-tail connector (Gp8), the tail tubular protein (Gp11), and the tail fiber protein (Gp17). These suggest that PpY1 may share similar functional characteristics with the T7-like phages.

### 3.5. Identification of Nonessential Genes of Phage PpY1

We initially attempted to assemble the PpY1 genome through homologous recombination or Gibson assembly in *E. coli* but failed, which may have resulted from the toxic effects of the phage proteins. Therefore, *S. cerevisiae* was selected as a host for phage genome assembly. A comprehensive gene deletion analysis of phage PpY1 was performed utilizing the TAR cloning technique (Figure 3B). This method allowed for the systematic removal of target genes from the phage genome. The wild-type genome and the modified versions, each lacking specific target genes, were amplified in segments via PCR and subsequently assembled within yeast. In total, 29 distinct genomes were successfully assembled to the pCAP01 vector (Table S1). The plasmids harboring the varied PpY1 genomes were extracted from yeast and introduced to the *P. aeruginosa* strain PAO1 by electroporation. However, this initial attempt did not yield any plaques on the agar plates. Given the potential interference of restriction–modification (RM) systems in DNA delivery, we conducted a search in the Restriction Enzyme Database (REBASE, http://rebase.neb.com, 10 June 2024) and identified a type I RM system (locus-tag: PA2732-PA2735; genes *PaePAIP*-*M. PaePAIP*) within the PAO1 genome. To circumvent this obstacle, an RM system-deficient strain, PAO1-KORM, was engineered by replacing the genes *PaePAIP*-*M. PaePAIP* with a gentamicin resistance gene (*gentR*). As illustrated in Figure S1, transferring the genome of a mutant phage into the PAO1-KORM led to the emergence of significantly more plaques compared to the strain PAO1 with the type-I RM system. The plasmids carrying either the wild-type or the partially deleted PpY1 genomes were then introduced into PAO1-KORM via electroporation. This approach resulted in the successful formation of plaques for 21 of the mutant PpY1 phage genomes (*gp01*, *gp02*, *gp03*, *gp04*, *gp05*, *gp06*, *gp08*, *gp09*, *gp10*, *gp11*, *gp12*, *gp13*, *gp14*, *gp16*, *gp17*, *gp21*, *gp23*, *gp26*, *gp34*, *gp55*, and *gp56*) (Figure S2), while no plaques were formed for the remaining eight gene deletions (*gp07*, *gp18*, *gp24*, *gp27*, *gp31*, *gp32*, *gp42*, *gp57*) (Figure S2). The plaques were isolated and their authenticity was verified by PCR and Sanger sequencing. To ascertain the genetic stability of the mutant phages, they were passaged through 10 generations within the host. Post-passaging, the genomes were extracted and their integrity was verified through PCR (Figure S3) and *Eco*R V restriction enzyme digestion (Figure S4). The findings indicated that the individual deletions of the 21 genes did not adversely impact the phage assembly process or genetic stability, suggesting that these genes are not essential for phage propagation under the tested conditions.

**Figure 4:**
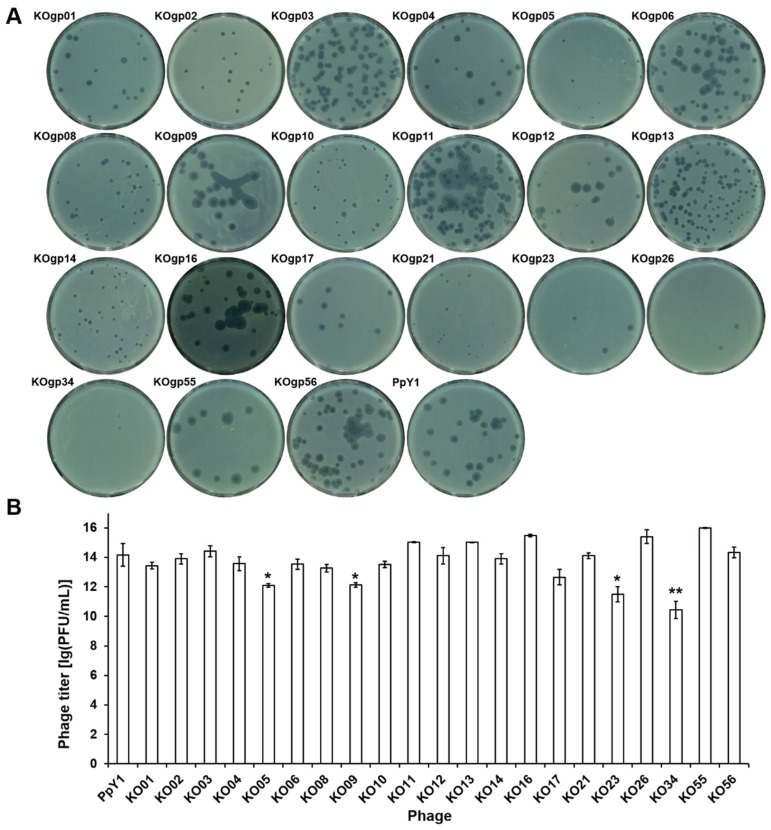
Analysis of phage plaques and titers. (**A**) Plaque morphology of mutant phages. (**B**) Phage titer of mutant phages. Error bars indicate the standard deviation (SD). *, *p* < 0.05; **, *p* < 0.01.

However, notable variations in both the morphology of the phage plaques and the phage titers were discerned among the mutant strains (Figure 4A). Specifically, the plaque sizes of PpY1-KOgp02, PpY1-KOgp05, PpY1-KOgp08, PpY1-KOgp10, and PpY1-KOgp14 were reduced in comparison to the wild-type phage PpY1. In contrast, the plaques of phages PpY1-KOgp21 and PpY1-KOgp34 were smaller, indicating a potential critical role for these genes in plaque formation. Yield measurements revealed that the phage titers of PpY1-KOgp05, PpY1-KOgp09, PpY1-KOgp17, PpY1-KOgp23, and PpY1-KOgp34 were significantly lower than that of the wild-type PpY1 (Figure 4B). This marked reduction or elevation in titer suggests that these phage genes may exert a substantial influence on the phage’s replication cycle or its ability to efficiently infect host cells. Notably, there was no strict correspondence between the titer and the size of the plaques. Although the deletion of gp05 and gp23 reduced both the size of plaques and phage titers, PpY1-KOgp21 and PpY1-KOgp23 retained a high titer and lytic activity, respectively.

### 3.6. Antibacterial Curve of Mutant Phages

**Figure 5:**
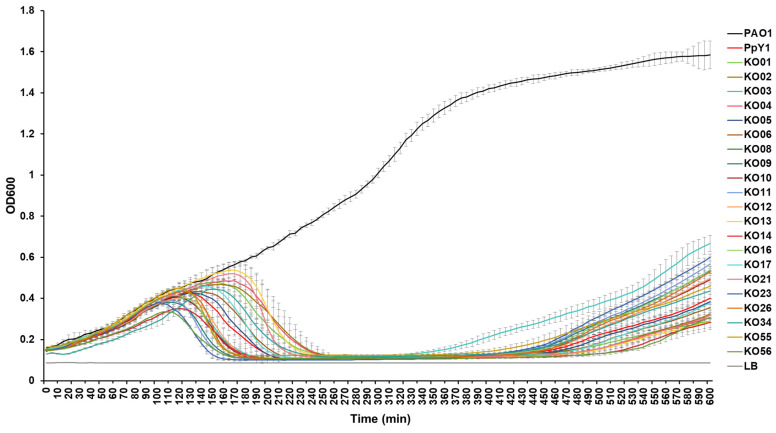
Lysis kinetics of PpY1 and the gene-knockout mutant phages at MOI of 0.01 on the *P. aeruginosa* strain PAO1. Error bars indicate the standard deviation (SD). The figure on the upper left shows the lysis kinetics of PpY1 and the mutants in 10 h. The area in the red box is enlarged.

Subsequently, we conducted a comparative analysis of the lysis kinetics of PpY1 and the gene-knockout mutant phages at a multiplicity of infection (MOI) of 0.01 in liquid medium (Figure 5). The optical density at 600 nm (OD_600_), which serves as a proxy for bacterial growth, exhibited distinct patterns for the wild-type phage PpY1 and its mutants. The OD_600_ value of the *P. aeruginosa* culture began to decrease at approximately 130 min after infection with wild phage PpY1, indicating the onset of lysis. Among the mutant phages, notable variations were observed. The OD_600_ value of the PAO1 culture infected with phages PpY1-KOgp08, PpY1-KOgp11, PpY1-KOgp23, and PpY1-KOgpPpY1-KOgp55 started to decline earlier, at around 110 min, suggesting a faster lysis initiation compared to PpY1 and a role of Gp08, Gp11, Gp23 and Gp56 in modulating the timing of lysis. Interestingly, the phage PpY1-KOgp56-infected culture displayed the lowest OD600 value of all, implying it achieved the most efficient lysis. In contrast, the lysis of PAO1 by phages PpY1-KOgp04, PpY1-KOgp13, PpY1-KOgp17, PpY1-KOgp21, and PpY1-KOgp34 was notably delayed, occurring at around 160 min, which is later than the wild-type PpY1. The delayed cell lysis by PpY1-KOgp21 underscored the relationship of Gp21 with cell lysis capacity. Gp04, Gp13, Gp17, Gp21, and Gp56 may be associated with the phage replication cycle, since no significant differences were found in the sizes of plaques between PpY1-KOgp13 and PpY1 (Figure 4).

### 3.7. Construction of Genome Reduced Phage

**Figure 6:**
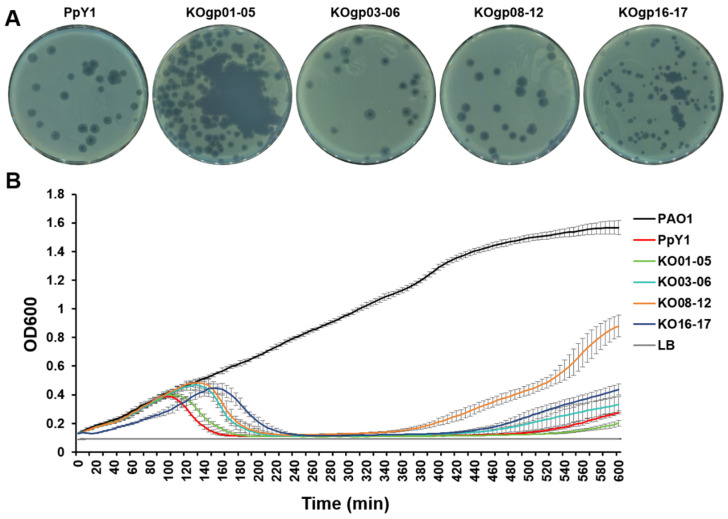
Plaque morphology and lysis kinetics of reduced phages. (**A**) Plaque morphology of reduced phages. (**B**) Lysis kinetics for wild and mutant phages at MOI of 0.01 on the *P. aeruginosa* strain PAO1. Each value is the mean of three different cultures ± SD.

After a thorough examination of the individual gene effects on phage PpY1, we proceeded to perform multiple deletions of nonessential genes within the PpY1 genome. Our sequence analysis revealed that a significant portion of the genes located in the upstream region of the PpY1 genome were nonessential for plaque formation. Given the consecutive arrangement and nonessential nature of the genes *gp01*-*gp06* and *gp08*-*gp12* and *gp16*-*gp17*, we focused our multiple gene deletion efforts on these genomic regions. The genes *gp01*-*gp03*, *gp04*-*gp05*, *gp06*-*gp12*, and *gp16*-*gp17* are situated within four separate operons. To minimize the potential negative impact on the expression of the crucial *gp07* gene, we strategically designed four distinct multiple gene deletion versions: KOgp01-05 (1351 bp deleted), KOgp03-06 (1249 bp deleted), KOgp08-12 (1188 bp deleted), and KOgp16-17 (641 bp deleted). In the KOgp03-06 version, the essential gene *gp07* and the downstream *gp08*-*gp12* were arranged to be co-transcribed with *gp02* to ensure proper regulation. As depicted in Figure 6A, the successful transfer of PpY1 genomic DNA with targeted deletions of *gp01*-*05*, *gp03*-*06*, *gp08*-*12*, or *gp16*-*17* into the strain PAO1-KORM resulted in the formation of plaques. The genomes of phages PpY1-KOgp01-05, PpY1-KOgp03-06, PpY1-KOgp08-12 and PpY1-KOgp16-17 were extracted and verified through *Eco*R V restriction enzyme digestion (Figure S5). This outcome confirms the successful assembly and functionality of the modified phages.

The plaque sizes produced by PpY1-KOgp16-17 were slightly smaller compared to those of the wild-type phage PpY1, while the other three mutant phages exhibited no significant differences in plaque size. In terms of phage titers, PpY1-KOgp01-05, PpY1-KOgp03-06, PpY1-KOgp08-12, PpY1-KOgp16-17, and PpY1 were quantified at 10^14^ PFU/mL, 10^14^ PFU/mL, 10^13^ PFU/mL, 10^12^ PFU/mL, and 10^13^ PFU/mL, respectively. We then determined the host range of genome-reduced phages to the 26 *P. aeruginosa* strains isolated from various environments. The host ranges of phages PpY1-KOgp03-06, PpY1-KOgp08-12, and PpY1-KOgp16-17 have been found to be more restricted compared to the wild-type PpY1. In the case of *P. aeruginosa* PA-DKB15-QH, obscured plaques were observed upon infection with PpY1 and PpY1-KOgp01-05, whereas no plaques were detected with PpY1-KOgp03-06, PpY1-KOgp08-12, and PpY1-KOgp16-17 (Table 2). Similarly, when *P. aeruginosa* YP-39 was infected, PpY1-KOgp03-06 and PpY1-KOgp08-12 failed to produce plaques, while PpY1, PpY1-KOgp01-05, and PpY1-KOgp16-17 exhibited clear plaque formation. These observations suggest that the deleted genes may play a role in determining the host range of phage PpY1.

The lysis kinetics of PpY1 and the genome reduced phages PpY1-KOgp01-05, PpY1-KOgp03-06, PpY1-KOgp08-12, and PpY1-KOgp16-17 were evaluated on the PAO1 strain at an MOI of 0.01 in liquid medium (Figure 6B). With the exception of PpY1-KOgp01-05, which displayed lysis kinetics comparable to the wild-type, the other genome-reduced phages showed a noticeable delay in cell lysis, suggesting that multiple deletion of the genes may influence replication of phages or the timing of the lysis process. Based on the multiple gene deletion results obtained, we attempted to construct a PpY1 genome by simultaneously removing *gp01*-*gp06*, *gp08*-*gp12*, and *gp16*-*gp17* (3600 bp) but no plaques were obtained.

## 4. Discussion

*P. aeruginosa* has emerged as a significant opportunistic pathogen responsible for substantial morbidity and mortality in recent decades [40,41,42]. Given the rise in antibiotic resistance, the highly specific host range and low side-effects from the eukaryotic organisms of bacteriophages offered unique advantages and considerable potential in the treatment of multi-drug resistant bacterial infections [43]. In this study, we successfully isolated a lytic phage, PpY1, which exhibits a broad host range against various *P. aeruginosa* strains, including multiple clinical isolates. Through extensive physiological and genomic characterization we determined that the phage PpY1 is a novel lytic phage classified within the genus *Phikmvvirus* and could tolerate a wide range of pH and temperature fluctuations. Phage PpY1 shares morphological and genomic structural similarities with the T7-like phage φKMV (Figure 1B and Figure 3). The distinct halos surrounding the plaques formed by PpY1 are indicative of its capacity to depolymerize exopolysaccharides and degrade biofilms, a trait that is desirable for therapeutic applications [44,45,46]. Furthermore, the cell lytic patterns and absence of toxins or antibiotic resistance genes within the PpY1 genome underscore its potential in phage therapy, which aligns with the characteristics of other φKMV-like phages [47,48,49].

The use of phages to combat infections is often limited by their narrow host specificity, a challenge that could potentially be overcome through genetic modifications. However, due to the limited understanding of phage genes and their genomic architecture, phage genome engineering remains challenging. In particular, there has been little advancement in the engineering of φKMV-like *P. aeruginosa* phages. While methods for assembling and engineering phage genomes in yeast and *E. coli* have been developed [30], their efficacy varies among different phages. Our research suggests that the success of phage genome engineering is significantly influenced by the presence of toxic elements within the phage genome and the host’s antiviral defense mechanisms, as evidenced by the difficulties in assembling the PpY1 genome in *E. coli* and the low efficiency of reactivating the genome in the wild-type *P. aeruginosa* PAO1. *P. aeruginosa* is known to possess several antiviral defense systems, including CRISPR-Cas system [50], RM systems [29], and Wadjet systems [51]. It has been reported that deletion of RM systems can increase the rebooting of phage genomes in *P. aeruginosa* [29]. In this study, we developed a practical approach for the efficient assembly and engineering of the φKMV-like phage PpY1 genome in yeast, followed by direct phage rescue in an RM system-deficient strain of *P. aeruginosa*.

The identification of essential and nonessential genes is a crucial step in the rational engineering of phage genomes. Recently, Yuan et al. developed a CRISPR/Cas9-based iterative phage genome reduction (CiPGr) approach, which randomly eliminates nonessential genes, achieving a genome reduction of 8–23% in four different phages [52]. A method combining homologous recombination with CRISPR-Cas13a was also developed for genome engineering of *P. aeruginosa* phage [24]. However, CRISPR-Cas system-based phage genome editing tools require construction of spacer library or targeting plasmids carrying selection marker and homology arms, which makes the workflow time consuming. In our study, we employed a direct and targeted approach using TAR cloning to in-frame delete specific genes in high efficiency, creating 28 PpY1 mutant phages and identifying 21 nonessential genes. While the individual deletion of these nonessential genes did not impact plaque formation, some affected plaque size, phage titer, or the timing of cell lysis. Notably, the deletion of genes *gp16*, *gp26*, or *gp55* significantly increased the phage titers, whereas the deletion of *gp08*, *gp11*, *gp23*, or *gp56* resulted in earlier bacterial lysis. The functions of these genes warrant further investigations. Phage genomes often contain numerous genes with unknown functions, and their regulatory mechanisms and genomic structures remain largely unclear, which hindered genome modifications for therapeutic applications. To explore the effects of deleting adjacent nonessential genes on phage functionality, we deleted four genomic regions (*gp01*-*gp05*, *gp03*-*gp06*, *gp08*-*gp12*, and *gp16*-*gp17*). These deletions showed no effects on plaque formation. However, we observed a significant reduction in titers for PpY1-KOgp16-17 and delayed cell lysis time for PpY1-KOgp03-06, PpY1-KOgp08-12, and PpY1-KOgp16-17. Interestingly, the individual deletion of *gp05* severely impacted the phage titers, but the simultaneous deletion of regions containing *gp05* (*gp01*-*gp05* and *gp03*-*gp06*) did not significantly affect titers. It is possible that gene deletions influence the phage genome structure, thereby affecting phage propagation and DNA encapsulation, leading to reduced titers and delayed bacterial cell lysis. Nonetheless, elucidation of the nonessential genes enabled a strategic and informed approach to genome modification, such as the incorporation of antibacterial elements, which could significantly enhance PpY1’s potential therapeutic and environmental disinfection purposes.

In summary, we isolated a lytic *P. aeruginosa* phage PpY1 from fecal sample. The broad host range, lytic capabilities, and biofilm-degrading properties of PpY1, along with its genetic safety profile, highlight the potential of PpY1 as a candidate for phage therapy against *P. aeruginosa* infections. A feasible approach was established to manipulate its genome in high efficiency. Systematic gene deletion identified 21 nonessential genes in the genome of PpY1. Further studies will focus on elucidating the specific functions of essential genes with unknown functions and the minimal genetic requirements for PpY1. These efforts may yield profound understanding into the intricacies of phage-host dynamics and lay the groundwork for the advancement of more potent phage-based treatment modalities.

## Figures and Tables

**Table 1:** Lysis activity of phage PpY1 against *P. aeruginosa* isolates.

Host Strains	PpY1	KOgp01−05	KOgp03−06	KOgp08−12	KOgp16−17	Origin
*P. aeruginosa* PAO1	+	+	+	+	+	Model strain
*P. aeruginosa* PA04	−	−	−	−	−	Mink lung
*P. aeruginosa* 130726.ZN	−	−	−	−	−	Feed for sick mink
*P. aeruginosa* YP−35	−	−	−	−	−	Duck embryo
*P. aeruginosa* PA−L27	−	−	−	−	−	Yolk of chicken embryo
*P. aeruginosa* C3SL	−	−	−	−	−	Feed for mink
*P. aeruginosa* PA−DANWO6	−	−	−	−	−	Duck’s nest
*P. aeruginosa* YP−2	−	−	−	−	−	Duck embryo
*P. aeruginosa* 111203	+	+	+	+	+	Mink lung
*P. aeruginosa* D.11092618	−	−	−	−	−	Mink lung
*P. aeruginosa* WD01	−	−	−	−	−	Mink lung
*P. aeruginosa* DC	−	−	−	−	−	Mink lung
*P. aeruginosa* SN03	+	+	+	+	+	Sheep lung
*P. aeruginosa* PA−631	+	+	+	+	+	Mink lung
*P. aeruginosa* PA−4	+	+	+	+	+	Chicken organs
*P. aeruginosa* PA−LSJ4	+	+	+	+	+	Feed for sick chickens
*P. aeruginosa* F2303	−	−	−	−	−	Human sputum
*P. aeruginosa* YP−25	+	+	+	+	+	Duck embryo
*P. aeruginosa* PA−XJ24030	−	−	−	−	−	Human pathogen isolated from antimicrobial disks
*P. aeruginosa* PA−JF4	−	−	−	−	−	Chicken lung
*P. aeruginosa* PA−DKB15−QH	*	*	−	−	−	Yolk of chicken embryo
*P. aeruginosa* D.11112309		+	+	+	+	Mink lung
*P. aeruginosa* YP−39	+	+	−	−	+	Duck embryo
*P. aeruginosa* YP−26	−	−	−	−	−	Duck embryo
*P. aeruginosa* PA−SX8−F3	−	−	−	−	−	Chicken fecal
*P. aeruginosa* PA−XJ230522054	−	−	−	−	−	Human pathogen isolated from antimicrobial disks
*P. aeruginosa* PA−XJ17030	−	−	−	−	−	Human pathogen isolated from antimicrobial disks

+, strong lytic activity; *, weak lytic activity; −, no lytic activity.

**Table 2:** Gene annotations of phage PpY1.

Gene	Strand	Size (bp)	Start	Stop	Function of Encoded Protein
*gp58*	−	213	380	168	hypothetical protein
*gp01*	+	285	1907	2191	hypothetical protein
*gp02*	+	228	2191	2418	hypothetical protein
*gp03*	+	540	2429	2968	hypothetical protein
*gp04*	+	105	3031	3135	hypothetical protein
*gp05*	+	120	3138	3257	hypothetical protein
*gp06*	+	369	3336	3704	hypothetical protein
*gp07*	+	228	3691	3918	hypothetical protein
*gp08*	+	186	3915	4100	hypothetical protein
*gp09*	+	180	4097	4276	hypothetical protein
*gp10*	+	282	4276	4557	hypothetical protein
*gp11*	+	261	4557	4817	hypothetical protein
*gp12*	+	288	4819	5106	hypothetical protein
*gp13*	+	423	5184	5606	hypothetical protein
*gp14*	+	360	5675	6034	hypothetical protein
*gp15*	+	855	6037	6891	DNA-binding protein
*gp16*	+	540	7250	7789	hypothetical protein
*gp17*	+	114	7794	7907	hypothetical protein
*gp18*	+	99	7904	8002	hypothetical protein
*gp19*	+	825	7975	8799	DNA primase
*gp20*	+	1269	8768	10,036	DNA helicase/AAA family ATPase
*gp21*	+	621	10,026	10,646	hypothetical protein
*gp22*	+	948	10,646	11,593	phage-associated ATP-dependent DNA ligase
*gp23*	+	285	11,590	11,874	hypothetical protein
*gp24*	+	321	11,871	12,191	hypothetical protein
*gp25*	+	2427	12,188	14,614	DNA-directed DNA polymerase
*gp26*	+	312	14,611	14,922	hypothetical protein
*gp27*	+	1050	14,977	16,026	hypothetical protein
*gp28*	+	942	16,026	16,967	5′−3′ exonuclease
*gp29*	+	441	16,957	17,397	endonuclease VII
*gp30*	+	1047	17,394	18,440	3′−5′exonuclease
*gp31*	+	372	18,450	18,821	hypothetical protein
*gp32*	+	351	18,814	19,164	hypothetical protein
*gp33*	+	2448	19,173	21,620	DNA−directed RNA polymerase
*gp34*	+	252	21,805	22,056	hypothetical protein
*gp35*	+	474	22,056	22,529	GNAT family N−acetyltransferase
*gp36*	+	297	22,474	22,770	virion structural protein
*gp37*	+	1533	22,782	24,314	Head-to-tail connector protein
*gp38*	+	969	24,318	25,286	capsid assembly protein
*gp39*	+	1008	25,339	26,346	major capsid protein
*gp40*	+	555	26,443	26,997	non-contractile tail tubular protein A
*gp41*	+	2481	27,000	29,480	Non-contractile tail tubular protein B
*gp42*	+	546	29,480	30,025	hypothetical protein
*gp43*	+	2697	30,025	32,721	baseplate hub structural protein/lysozyme R
*gp44*	+	4014	32,725	36,738	DNA ejectosome component, peptidoglycan lytic exotransglycosylase
*gp45*	+	756	36,740	37,495	tail fiber protein A
*gp46*	+	459	37,495	37,953	tail fiber protein B
*gp47*	+	906	37,946	38,851	tail fiber protein C
*gp48*	+	606	38,855	39,460	tail fiber protein D
*gp49*	+	306	39,460	39,765	terminase small subunit
*gp50*	+	1806	39,775	41,580	terminase large subunit
*gp51*	+	201	41,577	41,777	holin
*gp52*	+	483	41,774	42,256	endolysin
*gp53*	+	330	42,214	42,543	phage lambda Rz-like lysis protein
*gp54*	+	114	42,518	42,631	phage lambda Rz1-like protein
*gp55*	+	315	42,633	42,947	hypothetical protein
*gp56*	+	195	42,997	43,191	hypothetical protein
*gp57*	−	213	43,727	43,515	hypothetical protein

## Data Availability

The complete genome sequence of phage PpY1 was deposited into GenBank, and the accession number is PQ463998. Additional results have been included in Supplementary Materials.

## Supplementary Materials

The following supporting information can be downloaded at: https://www.mdpi.com/article/10.3390/microorganisms12112346/s1, Figure S1: Plaques of PpY1-KOgp03 generated by transferring pCAP01- PpY1-KOgp03 into PAO1 (A) and PAO1-KORM (B); Figure S2: Plaques of phage PpY1 and mutant phages generated by transferring assembled genomes into PAO1-KORM; Figure S3: Verification of mutant phage genomes by PCR. The genome of PpY1 was set as control. The amplified fragments were further validated by Sanger-sequencing; Figure S4: Restriction analysis of the genomes of mutant phages. The genomic DNA was digested by *Eco*R V. M, DNA marker; Figure S5: Restriction analysis of genome reduced phages. The genomic DNA was digested by *Eco*R V. M, DNA marker; Table S1: Primers used in this study; Table S2: Plasmids constructed in this study.
