# Supplementary material for: Isolation, Characterization, and Genome Engineering of a Lytic Pseudomonas aeruginosa Phage
Source: Microorganisms. 2024 Nov 16;12(11):2346. doi: 10.3390/microorganisms12112346 (PMC11596505; doi:10.3390/microorganisms12112346)
Supplement: Supplementary file 1 [file microorganisms-12-02346-s001.zip › microorganisms-3297554-supplementary.pdf]

Supplementary materials

Isolation, characterization and genome engineering of a lytic *Pseudomonas aeruginosa* phage

Xiaomei Cong,[1] Shuang Zhao,[1] Qing Zhang,[2] Shuo Liu,[1] Youming Zhang [1]* and Fu Yan [1]*

[1] *State Key Laboratory of Microbial Technology, Shandong University, Qingdao 266237, China*

[2] *Institute of Animal Science and Veterinary Medicine, Shandong Academy of Agricultural Sciences, Jinan 250000, China*

* Corresponding author: zhangyouming@sdu.edu.cn and fuyan@sdu.edu.cn

Table S1. Primers used in this study.

Primer	Sequence (5'->3')
PpY1 repeat-F	CCCGACTCCGACACGCCCAC
PpY1 Frag1-R	ACGCGGCGTGGTTTACTGCGGCGCGTGCCTTACGCTTAGCCTCTGC AACTTGGTCGGCA
PpY1 Frag2-F	TGCCGACCAAGTTGCAGAGGCTAAGCGTAAGGCACGCGCCGCAGT AAACCACGCCGCGT
PpY1 Frag2-R	TGTCGGATGAAGATTTAAGGCCGGAGTAGGGAAGGGTCGTCAGGC GTTCGAGGCGTGCG
PpY1 Frag3-F	CGCACGCCTCGAACGCCTGACGACCCTTCCCTACTCCGGCCTTAAA TCTTCATCCGACA
PpY1 Frag3-R	GATGCACTTGCTCCTATGTGGGTGACATAATCCGCCTCAGCAGAAG AACGCCCAGGAGT
PpY1 Frag4-F	ACTCCTGGGCGTTCTTCTGCTGAGGCGGATTATGTCACCCACATAG GAGCAAGTGCATC
PpY1 Frag4-R	CCAAGCTCTTGGGAAGCACGTTGCGATTCCGCCATCAGTTGCCTCC TTTAGGCGTGGCG
PpY1 Frag5-F	CGCCACGCCTAAAGGAGGCAACTGATGGCGGAATCGCAACGTGCT TCCCAAGAGCTTGG
PpY1 Frag5-R	CGAATCAGGGTGTAACGTCCCTTTACTTCGGTCTTAAACATTTTTCA GCTCCGCCTGTA
PpY1 Frag6-F	TACAGGCGGAGCTGAAAAATGTTTAAGACCGAAGTAAAGGGACGT TACACCCTGATTCG
PpY1 repeat-R	CCAGCCACTCCCGCAACCAC
KOgp01-Frag1-1-R	AGTTCATTCACACGTGGCATCGTGCAACCTCCTACTAGGTCTGTCT CTTGAAT
KOgp01-Frag1-2-F	ATTCAAGAGACAGACCTAGTAGGAGGTTGCACGATGCCACGTGTG AATGAACT
KOgp02-Frag1-1-R	TTGGAGATTGCGTTGGTCATTACAAACCCTTCCCTTGCGA
KOgp02-Frag1-2-F	TCGCAAGGGAAGGGTTTGTAATGACCAACGCAATCTCCAA
KOgp03-Frag1-1-R	TAGCAGGCGTTCGACTTGAGGGTATGTACCTCAGATTGAT
KOgp03-Frag1-2-F	ATCAATCTGAGGTACATACCCTCAAGTCGAACGCCTGCTA
KOgp04-Frag1-1-R	GGCGATCCAGAAGTCCATGGCGTGTTTCTCCAGTTGTCAT
KOgp04-Frag1-2-F	ATGACAACTGGAGAAACACGCCATGGACTTCTGGATCGCC
KOgp05-Frag1-1-R	CGCCAGGGCCATGACTTGAGGGTCACTCCCCCTTCGCCTG
KOgp05-Frag1-2-F	CAGGCGAAGGGGGAGTGACCCTCAAGTCATGGCCCTGGCG
KOgp06-Frag1-1-R	TAGGGATCACGCGAGGACATCGTGCAACCTCCTGGTTTAGTTCTCG CTTGTGA
KOgp06-Frag1-2-F	TCACAAGCGAGAACTAAACCAGGAGGTTGCACGATGTCCTCGCGT GATCCCTA
KOgp07-Frag1-1-R	GCTATGATGAGCCACTTCATCGTGCAACCTCCTTCACGCGAGGACA TGGTACG
KOgp07-Frag1-2-F	CGTACCATGTCCTCGCGTGAAGGAGGTTGCACGATGAAGTGGCTC ATCATAGC

KOgp08-Frag1-1-R	ACGACGTAGGTAGGATTCATCGTGCAACCTCCTTCATTTACGGGTCGGTAGCT
KOgp08-Frag1-2-F	AGCTACCGACCCGTAAATGAAGGAGGTTGCACGATGAATCCTACCTACGTCGT
KOgp09-Frag1-1-R	TACACGGTACGGACGGTCATCGTGCAACCTCCTTCATATGCGCTTGCTCCAGT
KOgp09-Frag1-2-F	ACTGGAGCAAGCGCATATGAAGGAGGTTGCACGATGACCGTCCGTACCGTGTA
KOgp10-Frag1-1-R	AGGATCAGTGCATCGGACATCGTGCAACCTCCTTTACTCACCCTCCTTCACGA
KOgp10-Frag1-2-F	TCGTGAAGGAGGGTGAGTAAAGGAGGTTGCACGATGTCCGATGCACTGATCCT
KOgp11-Frag1-1-R	TGGGTATGGGTACGGATCATCGTGCAACCTCCTTCAGTCGTCTCCTTGGACGA
KOgp11-Frag1-2-F	TCGTCCAAGGAGACGACTGAAGGAGGTTGCACGATGATCCGTACCCATACCCA
KOgp12-Frag1-1-R	CGCACCAGCCTTCGCCTTGGCTCAGTTCCTCGTCGATTTG
KOgp12-Frag1-2-F	CAAATCGACGAGGAACTGAGCCAAGGCGAAGGCTGGTGCG
KOgp13-Frag1-R	GGGCCTATTACGTGCGGGCCGCTGTAGCTCCTCAGTTAGG
KOgp13-Frag2-F	CCTAACTGAGGAGCTACAGCGGCCCGCACGTAATAGGCCC
KOgp14-Frag1-R	TAAAGGTCTTTGCACTGCATGGTGACTCCTCCTATGGTAT
KOgp14-Frag2-F	ATACCATAGGAGGAGTCACCATGCAGTGCAAAGACCTTTA
KOgp16-Frag1-R	TTCGCGATAAGCCATGTCATCGTGCAACCTCCTTCTAAATCCCTCAATTTG
KOgp16-Frag2-F	CAAATTGAGGGATTTAGAAGGAGGTTGCACGATGACATGGCTTATCGCGAA
KOgp17-Frag1-R	TCCTGAAGATGCCAGGTCATCGTGCAACCTCCTCACCTCACTCATGATCGGGT
KOgp17-Frag2-F	ACCCGATCATGAGTGAGGTGAGGAGGTTGCACGATGACCTGGCATCTTCAGGA
KOp18-Frag1-R	GAGTCTCGGCGCAGTGCCATCGTGCAACCTCCTTCATGCGGGTGCCGTAGGCG
KOp18-Frag2-F	CGCCTACGGCACCCGCATGAAGGAGGTTGCACGATGGCACTGCGCCGAGACTC
KOp21-Frag1-R	ACCACGTCGCGCTTGCTCATTTAGCTTGCATGATCTACAC
KOp21-Frag2-F	GTGTAGATCATGCAAGCTAAATGAGCAAGCGCGACGTGGT
KOp23-Frag2-1-R	TCCTCGGTGGGCATTCTCATTCATACCTTCACTCCAGGGT
KOp23-Frag2-2-F	ACCCTGGAGTGAAGGTATGAATGAGAATGCCCACCGAGGA
KOp24-Frag2-1-R	AGGATTCGGATAGTCGTCATTCATACTTCGACTCCTACCT
KOp24-Frag2-2-F	AGGTAGGAGTCGAAGTATGAATGACGACTATCCGAATCCT
KOp26-Frag2-R	GCCGGAGTAGGGAAGGGTCGTCACTGAATCACCTCCATAT
KOp26-Frag3-F	ATATGGAGGTGATTCAGTGACGACCCTTCCCTACTCCGGC
KOp27-Frag2-R	TCTTCCGATGGCAGGCGCATGCGTGGTCTCTCTCGTGTCG
KOp27-Frag3-F	CGACACGAGAGAGACCACGCATGCGCCTGCCATCGGAAGA

KOgp31-Frag3-1-R	GGTACACCAACAGATTTTACTCATGCTACCTCCTTAGTTGCTCA
KOgp31-Frag3-2-F	TGAGCAACTAAGGAGGTAGCATGAGTAAAATCTGTTGGTGTACC
KOgp32-Frag3-2-F	GGGAGAAAGGCAATGAGTAAAGGAGGTTGCACGATGGACCTGATACAGCAGCA
KOgp32-Frag3-1-R	TGCTGCTGTATCAGGTCCATCGTGCAACCTCCTTTACTCATTGCCTTTCTCCC
KPgp34-Frag3-R	CGCCAGATGGACTTGTTCATCGTGCAACCTCCTCTATCATATCTCCTATGCTG
KOgp34-Frag4-F	CAGCATAGGAGATATGATAGAGGAGGTTGCACGATGAACAAGTCCATCTGGCG
KOgp42-Frag4-R	TCCGCCATCAGTTGCCTCCTTCACACCCTCCTGTAGGTTT
KOgp42-Frag5-F	AAACCTACAGGAGGGTGTGAAGGAGGCAACTGATGGCGGA
KOgp55 Frag6-2-F	CCTTGAATCAAGGAGATTGAGCTTCAGCCCGTCCAACCTG
KOgp56 Frag6-1-R	GCTCGGCTTGAGCTACTCGAGGTTCAGTTCCTTGTGTTAT
KOgp56-Frag6-2-F	ATAACACAAGGAACTGAACCTCGAGTAGCTCAAGCCGAGC
PpY1-KOgp57-Frag1-2-F	TACGCATCCTGGCGGGAGTGCCTCTGTGCCTCCTGTGGTG
PpY1-KOgp57-Frag1-1-R	CACCACAGGAGGCACAGAGGCACTCCCGCCAGGATGCGTA
KOgp01-chkL-F	ATTGAACAAGGCCAGGCAGA
KOgp02-chkR-R	ATGGCTTCGTCCAGTTCCTT
KOgp04-chkR-R	CCACAACATGCGCAGTTCAT
KOgp05-chkL-F	AGCTTCAAACAACGCCTGCA
KOgp06-chkR-R	GGTCGGGTTATGGCTTCAAT
KOgp07-chkL-F	ATGGCTGGATCAACGCTGAA
KOgp10-chkR-R	CGCGATCAGGATCAGTGCAT
KOgp11-chkL-F	AAGGCGAGTACCTCAAACTC
KOgp12-chkR-R	AGCTCCTCAGTTAGGTGGTT
KOgp13-chkL-F	AGCCTTCCACCGTGGCCATT
KOgp14-chkR-R	GTACGCTGAACATGTCCGAT
KOgp16-chkL-F	ATTACATGGCGCAGGACCTG
KOgp18-chkR-R	TGGCCGACTGCCAGGGATTG
KOgp21-chkL-F	AAATCCACCTGGGCCGTCTC
KOgp21-chkR-R	CGCGATGATGCGATCCTCCA
KOgp23 chkL-F	AACTATGGACGCATCGCCGA
KOgp23 chkR-R	GGTCAGTTGGTACAGGGAGA
KOgp24-chkR-R	CGAACATGGCATTGTGAGCT
KOgp26-chkL-F	ACATCATGGCCGACGCTGCC
KOgp26-chkR-R	CCGGTGGAGGTTTCGGACAT
KOgp41-chkL-F	AGAGGACTACGAAGGATTCT
KOgp42-chkR-R	GGTCAACCTGCTGGTTTCGT
KOgp56-chkR-R	CCAGTGGAGCACCCGACTAT
genta-KORM-F1	GCACACGTCAACTGTGGCTTGCCACTAAACATATTTGAATGAAGAGTTGAAGCGCTGATAGAAGGCACGAACCCAGTTGAC

genta-KORM-F2	TAATGGGCACGAATGGCCGGAGCGTATTTTCCCTATCAAGGCATCC CATGGCCACGGCCCGCACACGTCAACTGTGGCTT
genta-KORM-4-R1	GCCGTTCTGGCTTGGAATAATTTTCGCAGCACTGGAAACCAGCCCA ACAGTGTTTTCAGACCGTTCGTATAGCATACATTATAC
genta-KORM-4-R2	GCTTCCATGCGCTGAACACAATCCTCGAAATGACGGTCAAGAAAA TCGTCGAACTCCTCCGCCGTTCTGGCTTGGAATAA

Table S2. Plasmids constructed in this study.

**Plasmids**	**Characteristics**	**Sources**	**Genome rebooting**
pCAP01-PpY1-KOgp01	pCAP01 carrying PpY1 genome deleted *gp01*	This study	+
pCAP01-PpY1-KOgp02	pCAP01 carrying PpY1 genome deleted *gp02*	This study	+
pCAP01-PpY1-KOgp03	pCAP01 carrying PpY1 genome deleted *gp03*	This study	+
pCAP01-PpY1-KOgp04	pCAP01 carrying PpY1 genome deleted *gp04*	This study	+
pCAP01-PpY1-KOgp05	pCAP01 carrying PpY1 genome deleted *gp05*	This study	+
pCAP01-PpY1-KOgp06	pCAP01 carrying PpY1 genome deleted *gp06*	This study	+
pCAP01-PpY1-KOgp07	pCAP01 carrying PpY1 genome deleted *gp07*	This study	−
pCAP01-PpY1-KOgp08	pCAP01 carrying PpY1 genome deleted *gp08*	This study	+
pCAP01-PpY1-KOgp09	pCAP01 carrying PpY1 genome deleted *gp09*	This study	+
pCAP01-PpY1-KOgp10	pCAP01 carrying PpY1 genome deleted *gp10*	This study	+
pCAP01-PpY1-KOgp11	pCAP01 carrying PpY1 genome deleted *gp11*	This study	+
pCAP01-PpY1-KOgp12	pCAP01 carrying PpY1 genome deleted *gp12*	This study	+
pCAP01-PpY1-KOgp13	pCAP01 carrying PpY1 genome deleted *gp13*	This study	+
pCAP01-PpY1-KOgp14	pCAP01 carrying PpY1 genome deleted *gp14*	This study	+
pCAP01-PpY1-KOgp16	pCAP01 carrying PpY1 genome deleted *gp16*	This study	+
pCAP01-PpY1-KOgp17	pCAP01 carrying PpY1 genome deleted *gp17*	This study	+
pCAP01-PpY1-KOgp18	pCAP01 carrying PpY1 genome deleted *gp18*	This study	−

pCAP01-PpY1-KOgp21	pCAP01 carrying PpY1 genome deleted *gp21*	This study	+
pCAP01-PpY1-KOgp23	pCAP01 carrying PpY1 genome deleted *gp23*	This study	+
pCAP01-PpY1-KOgp24	pCAP01 carrying PpY1 genome deleted *gp24*	This study	−
pCAP01-PpY1-KOgp26	pCAP01 carrying PpY1 genome deleted *gp26*	This study	+
pCAP01-PpY1-KOgp27	pCAP01 carrying PpY1 genome deleted *gp27*	This study	−
pCAP01-PpY1-KOgp31	pCAP01 carrying PpY1 genome deleted *gp31*	This study	−
pCAP01-PpY1-KOgp32	pCAP01 carrying PpY1 genome deleted *gp32*	This study	−
pCAP01-PpY1-KOgp34	pCAP01 carrying PpY1 genome deleted *gp34*	This study	+
pCAP01-PpY1-KOgp42	pCAP01 carrying PpY1 genome deleted *gp42*	This study	−
pCAP01-PpY1-KOgp55	pCAP01 carrying PpY1 genome deleted *gp55*	This study	+
pCAP01-PpY1-KOgp56	pCAP01 carrying PpY1 genome deleted *gp56*	This study	+
pCAP01-PpY1-KOgp57	pCAP01 carrying PpY1 genome deleted *gp57*	This study	−

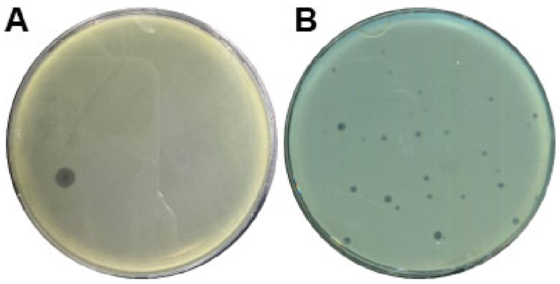

Figure S1 Plaques of PpY1-KOgp03 generated by transferring pCAP01- PpY1-KOgp03 into PAO1 (A) and PAO1-KORM (B).

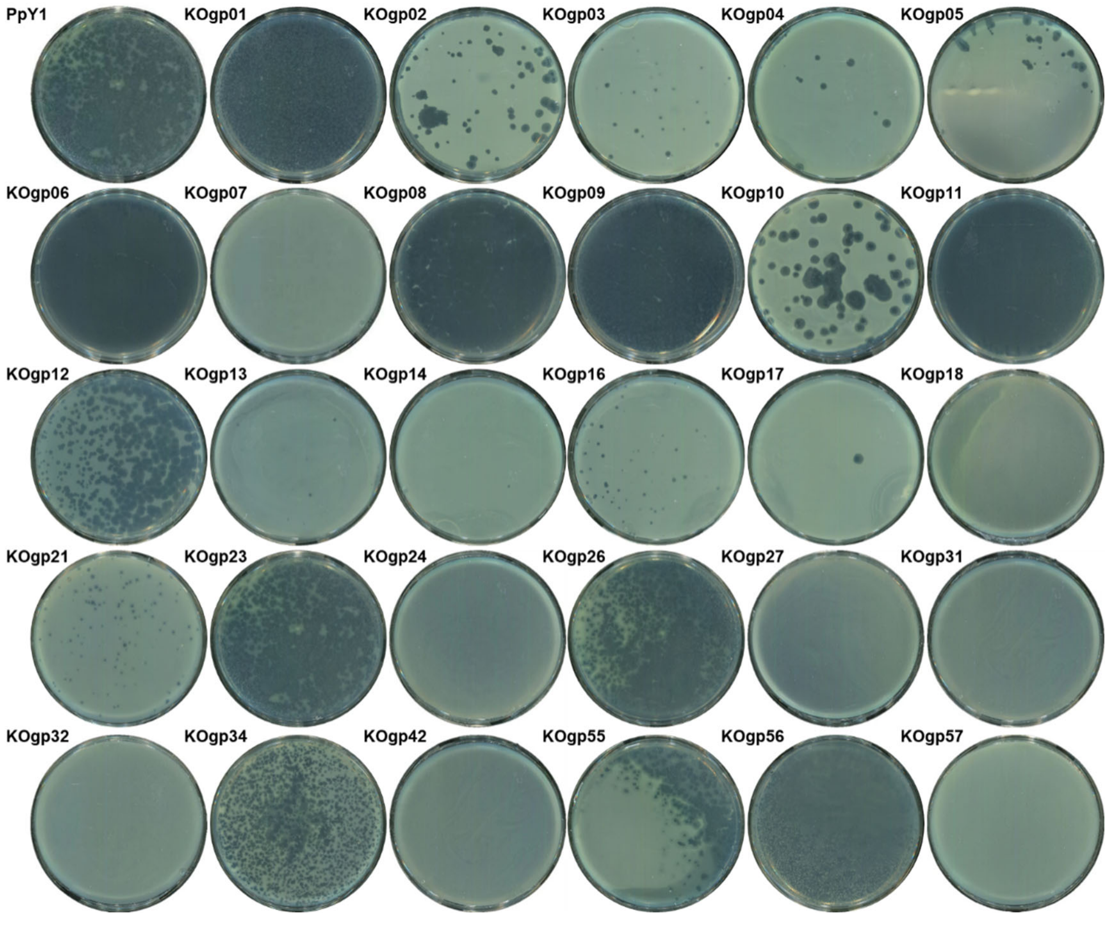

Figure S2 Plaques of phage PpY1 and mutant phages generated by transferring assembled genomes into PAO1-KORM.

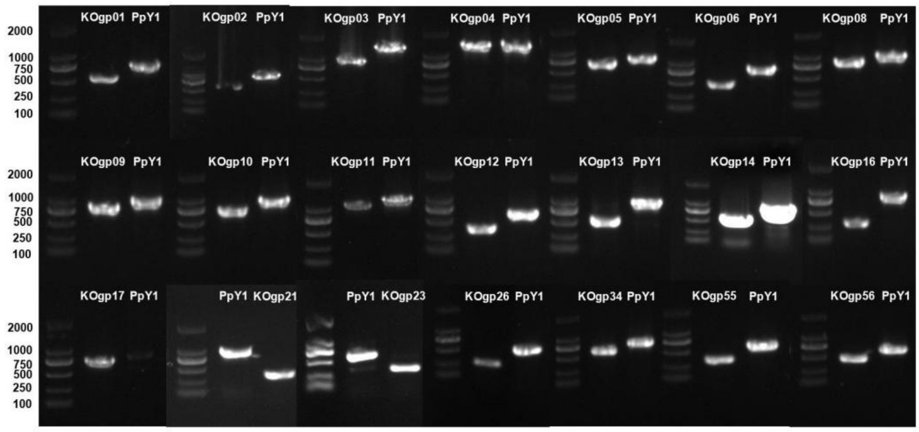

Figure S3 Verification of mutant phage genomes by PCR. The genome of PpY1 was set as control. The amplified fragments were further validated by Sanger-sequencing.

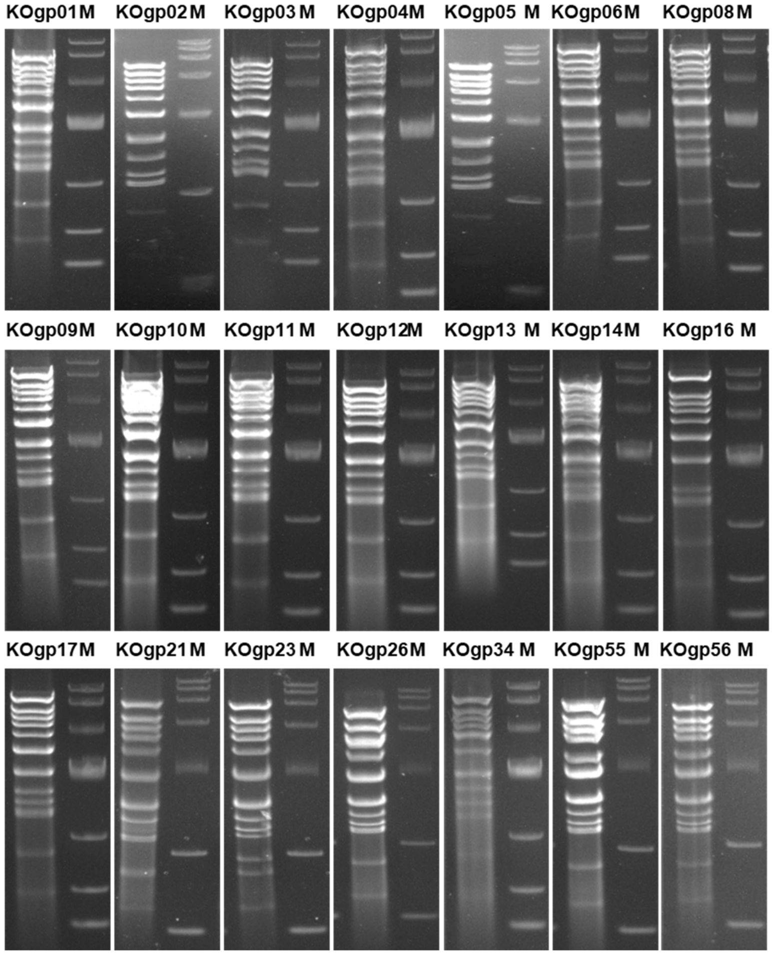

Figure S4 Restriction analysis of the genomes of mutant phages. The genomic DNA were digested by *Eco*R V. M, DNA marker.

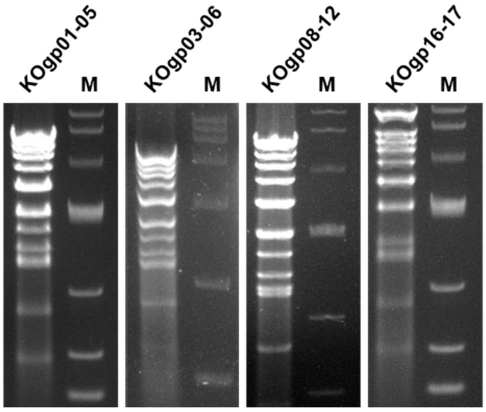

Figure S5 Restriction analysis of genome reduced phages. The genomic DNA were digested by *Eco*R V. M, DNA marker.
